# Optimal chemotherapy treatment for women with recurrent ovarian cancer

**DOI:** 10.3747/co.2007.148

**Published:** 2007-10

**Authors:** M. Fung-Kee-Fung, T. Oliver, L. Elit, A. Oza, H.W. Hirte, P. Bryson

**Affiliations:** * The Ottawa Hospital Regional Cancer Centre, Ottawa, Ontario; † Program in Evidence-Based Care, McMaster University, Hamilton, Ontario.; ‡ Juravinski Regional Cancer Centre, Hamilton, Ontario.; § Princess Margaret Hospital, Toronto, Ontario.; || Kingston General Hospital, Kingston, Ontario

**Keywords:** Chemotherapy, drug therapy, ovarian cancer, ovarian neoplasms, practice guideline, systematic review

## Abstract

**Question:**

What is the optimal chemotherapy treatment for women with recurrent ovarian cancer who have previously received platinum-based chemotherapy?

**Perspectives:**

Currently, standard primary therapy for advanced disease involves a combination of maximal cytoreductive surgery and chemotherapy with carboplatin plus paclitaxel or with carboplatin alone. Despite initial high response rates, a large proportion of patients relapse, resulting in a therapeutic challenge. Because these patients are not curable, the goal of therapy becomes improvement in both quality and length of life. The search has therefore been to find active agents for women with recurrent disease following platinum-based chemotherapy.

**Outcomes:**

Outcomes of interest included any combination of tumour response rate, progression-free survival, overall survival, adverse events, and quality of life.

**Methodology:**

The medline, embase, and Cochrane Library databases were systematically searched for primary articles and practice guidelines. The resulting evidence informed the development of clinical practice recommendations. The systematic review and recommendations were approved by the Report Approval Panel of the Program in Evidence-Based Care, and by the Gynecology Cancer Disease Site Group (dsg). The practice guideline was externally reviewed by a sample of practitioners from Ontario, Canada.

**Results:**

Thirteen randomized trials compared various chemotherapy regimens for patients with recurrent ovarian cancer.

In five of the thirteen trials in which 100% of patients were considered sensitive to platinum-containing chemotherapy, further platinum-based combination chemotherapy significantly improved response rates (two trials), progression-free survival (four trials), and overall survival (three trials) when compared with single-agent chemotherapy involving carboplatin or paclitaxel. Only two of these randomized trials compared the same chemotherapy regimens: carboplatin alone versus the combination of carboplatin and paclitaxel. Both trials were consistent in reporting improved survival outcomes with the combination of carboplatin and paclitaxel. In one trial, the combination of carboplatin and gemcitabine resulted in significantly higher response rates and improved progression-free survival when compared with carboplatin alone. Median survival with carboplatin alone ranged from 17 months to 24 months in four trials.

In eight of the thirteen trials in which 35%–100% of patients had platinum-refractory or -resistant disease, one trial reported a statistically significant 2-month improvement in overall survival with liposomal doxorubicin as compared with topotecan (15 months vs. 13 months, *p* = 0.038; hazard ratio: 1.23; 95% confidence interval: 1.01 to 1.50). In that trial, because of the limited clinical benefit and the unusual finding that a survival difference emerged only after a year of treatment with no corresponding improvement in the rate of response or of progression-free survival, the authors concluded that further confirmation by results from randomized trials were needed to establish the superiority of one agent over another in their trial. In one trial, topotecan was superior to treosulphan in patient progression-free survival by a span of approximately 2 months (5.4 months vs. 3.0 months, *p* < 0.001).

Toxicity was reported in all of the randomized trials, and although data on adverse events varied by treatment regimen, the observed adverse events correlated with known toxicity profiles. As expected, combination chemotherapy was associated with higher rates of adverse events.

**Practice Guideline:**

**Target Population**

This clinical recommendation applies to women with recurrent epithelial ovarian cancer who have previously received platinum-based chemotherapy. Of specific interest are women who have previously shown sensitivity to platinum therapy and those who previously were refractory or resistant to platinum-based chemotherapy. As a general categorization within what is actually a continuum, “platinum sensitivity” refers to disease recurrence 6 months or more after prior platinum-containing chemotherapy, and “platinum resistance” refers to a response to platinum-based chemotherapy followed by relapse less than 6 months after chemotherapy is stopped. “Platinum-refractory disease” refers to a lack of response or to progression while on platinum-based chemotherapy.

**Recommendations**

Although the body of evidence that informs the clinical recommendations is based on randomized trial data, those data are incomplete. Based on the available data and expert consensus opinion, the Gynecology Cancer dsg makes these recommendations:

- Systemic therapy for recurrent ovarian cancer is not curative. It is therefore recognized that each patient must be individually assessed to determine optimal therapy in terms of recurrence, sensitivity to platinum, toxicity, ease of administration, and patient preference. All suitable patients should be offered the opportunity to participate in randomized trials, if available.
- In the absence of contraindications, combination platinum-based chemotherapy should be considered for patients with prior sensitivity to platinum-containing chemotherapy. As compared with carboplatin alone, the combination of carboplatin and paclitaxel significantly improved both progression-free and overall survival.
- If combination platinum-based chemotherapy is not indicated, then a single platinum agent should be considered. Carboplatin has demonstrated efficacy across trials and has a manageable toxicity profile.
- If a single platinum agent is not being considered, then monotherapy with paclitaxel, topotecan, or pegylated liposomal doxorubicin are seen as reasonable treatment options.
- Some patients may be repeatedly sensitive to treatment and may benefit from multiple lines of chemotherapy.
- For patients with platinum-refractory or platinum-resistant disease, the goals of treatment should be to improve quality of life by extending the symptom-free interval, by reducing symptom intensity, and by increasing progression-free interval, and, if possible, to prolong life.
- With non-platinum agents, monotherapy should be considered because no advantage appears to accrue to the use of non-platinum-containing combination chemotherapy in this group of patients. Single-agent paclitaxel, topotecan, or pegylated liposomal doxorubicin have demonstrated activity in this patient population and are reasonable treatment options.
- No evidence either supports or refutes the use of more than one line of chemotherapy in patients with platinum-refractory or platinum-resistant recurrence. Many treatment options have shown modest response rates, but their benefits over best supportive care have not been studied in clinical trials.

## 1. QUESTION

What is the optimal chemotherapy treatment for women with recurrent ovarian cancer who have previously received platinum-based chemotherapy?

## 2. CHOICE OF TOPIC AND RATIONALE

In Canada, ovarian cancer is the fifth leading cause of cancer death among women and the leading cause of gynecologic cancer mortality 1. Estimates suggest that approximately 2400 new cases of ovarian cancer and 1550 deaths from the disease occurred in 2005, for a mortality-to-incidence ratio of 0.66 1.

Currently, standard primary therapy for advanced disease involves the combination of maximal cytoreductive surgery and chemotherapy with carboplatin plus paclitaxel or with carboplatin alone 2,3. Despite high initial response rates, a large proportion of patients relapse 4, resulting in a therapeutic challenge. Because these patients are not curable 5, the goal of therapy becomes improvement in both quality and length of life. The willingness of patients to undergo aggressive therapies for modest gains is growing, as has been documented for some other disease sites 6. This attitude has added a further dimension to the question of optimal treatment choice in this setting. The search has therefore been for active agents in women with recurrent disease after platinum-based chemotherapy.

One of the most frequently documented clinical surrogates for predicting response to chemotherapy in women with recurrent ovarian cancer has been the “platinum-free interval”—that is, the period of time from cessation of primary platinum-based chemotherapy to disease recurrence. Described in terms of platinum resistance or sensitivity, “responsiveness” is best characterized as a continuum. Increasing sensitivity to platinum is positively correlated with time from initial treatment to recurrence, such that disease that recurs more than 2 years after primary chemotherapy has a response rate to re-treatment with platinum approaching that associated with primary chemotherapy 7.

Before a patient is treated with platinum-based chemotherapy, it is impossible to assess whether she will or will not be sensitive to platinum. In addition, cancer in individual patients becomes increasingly resistant to platinum over time. However, the rate at which resistance develops is variable, and thus some women respond to multiple lines of platinum-based chemotherapy and some respond not at all.

Women with platinum-resistant disease have uniformly low response rates to chemotherapy. Further complicating the decision of what to offer these patients is the wide range of approaches to treatment among specialists dealing with this dilemma. The options range from using less toxic treatments as therapy for symptomatic disease, including single-agent chemotherapy or best supportive care, to using a range of multi-agent regimens in aggressive therapy for asymptomatic patients.

Generally, the disease can be defined as “platinum-resistant” if recurrence is diagnosed less than 6 months after platinum-based chemotherapy has been completed. “Platinum-sensitive” disease can be defined as recurrence 6 months or more after the end of platinum-based chemotherapy. Although these definitions are somewhat arbitrary (because platinum sensitivity should be regarded more as a continuum), the decision to establish a 6-month cut-off for defining platinum resistance and sensitivity is supported by the outcomes from other studies of platinum-pre-treated patients with ovarian cancer 8,9.

The present review of the evidence focuses on systemic chemotherapy; it does not evaluate intraperitoneal chemotherapy, hormonal therapy, or chemotherapy with bone marrow or stem-cell transplantation. The role of hormonal therapy in the treatment of recurrent ovarian cancer, although limited, is acknowledged to have been well addressed in published reviews 10,11. The present review summarizes the best available evidence on the optimal use of various chemotherapeutic agents in the treatment of platinum-sensitive and platinum-resistant recurrent ovarian cancer.

## 3. METHODS

### 3.1 Guideline Development

The present systematic review of the evidence was developed by Cancer Care Ontario’s Program in Evidence-Based Care (pebc) using the methods of the practice guidelines development cycle 12. Evidence was selected and extracted by one member of the pebc’s Gynecology Cancer Disease Site Group (dsg) and by a methodologist. All drafts of the text were reviewed, modified, and approved by the Gynecology Cancer dsg, and also by a Report Approval Panel (rap) of the pebc.

This review is a convenient and up-to-date source of the best available evidence on chemotherapy in the treatment of women with recurrent epithelial ovarian cancer. The body of evidence upon which the review is based primarily comprises data from randomized controlled trials (rcts). The systematic review and companion practice guideline developed by the Gynecology Cancer dsg are intended to promote evidence-based practice in Ontario, Canada. The pebc is editorially independent of Cancer Care Ontario and the Ontario Ministry of Health and Long-Term Care.

An evidence summary on the management of women with recurrent ovarian cancer was originally completed in 2001 by the Gynecology Cancer dsg 13. The present document replaces the 2001 report.

### 3.2 Literature Search Strategy

The medline (ovid: 1966 through March 2006), embase (ovid: 1988 through March 2006), Cochrane Library (ovid: Issue 1, 2006), the Canadian Medical Association Infobase, and the National Guidelines Clearinghouse were systematically searched for evidence relevant to this report. In addition, the abstracts published in the proceedings of the meetings of the American Society of Clinical Oncology (1997–2005) and the European Society for Medical Oncology (2002, 2004) were similarly searched. Reference lists of related papers and recent review articles were scanned for additional citations.

Searches of electronic databases combined the terms (ovarian neoplasms/ or ovarian.ti. and neoplasm:.mp. or cancer.mp.) with (neoplasm recurrence local/ or neoplasm metastasis/ or recurren:.mp. or relapse:.mp. or resistance.mp.) and (drug therapy/ or antineoplastic agents/ or chemotherapy.mp.) for these study designs: rcts, meta-analyses, practice guidelines, and systematic reviews.

### 3.3 Study Selection Criteria

Articles were selected for inclusion in this systematic review of the evidence if they were rcts that compared chemotherapeutic agents as part of second- or greater-line treatment for patients with recurrent epithelial ovarian cancer who had previously been treated with platinum-containing chemotherapy, and if they reported data on at least one of the following outcomes of interest: overall survival, progression-free survival, tumour response rate, adverse events, or quality of life (qol).

Practice guidelines, meta-analyses, or systematic reviews explicitly based on randomized trials related to the guideline question were also eligible for inclusion in the systematic review of the evidence.

Articles were excluded from the systematic review of the evidence if they were written in a language other than English or if they included the use of intraperitoneal chemotherapy, hormonal therapy, or chemotherapy with bone marrow or stem-cell transplantation.

## 4. RESULTS

### 4.1 Literature Search Results

Thirteen randomized trials comparing various chemotherapy regimens for women with recurrent ovarian cancer were identified and deemed eligible for inclusion in the systematic review of the evidence 14–26. In the event of multiple publications per trial, only the most recent publication was cited. Ten trials were fully published 14–18,20,22–24,26, and three trials were reported as abstracts from conference proceedings 19,21,25. Table I presents the literature search results and select trial characteristics.

### 4.2 Trial Characteristics

To be eligible to participate in the randomized trials, patients had to have recurrent ovarian cancer after prior first-line 14,16–18,21–23,25,26 or prior first- or second-line treatment 15,19,20,24 with platinum-containing chemotherapy. Where reported, patients were stratified by age 18,20, presence of ascites 18, type of prior chemotherapy 14,20, chemotherapy-free interval 20,24, number of lines of treatment 20,24, bi-dimensionally measurable disease 14, performance status 20,24, platinum-free interval 14,16–18,20,21, presence of bulky disease exceeding 5 cm 17,21, or treatment center 16,20,24.

The median age of the patients ranged from a low of 54 years to a high of 61 years 14–18,20–24. Eight trials included patients over the age of 70 years 14–18, 21,23,24, and four trials included patients who were more than 80 years of age 14,17,18,24. With the exception of one trial 18, information on patient height, weight, body mass index, or menopausal status was not reported. In the one trial that reported those data 18, the mean weight of the patients was approximately 66 kg, and the mean body surface was 1.7 m^2^.

As can be seen in Table I, various chemotherapeutic regimens were investigated across the thirteen trials. Carboplatin, paclitaxel, and topotecan were the most commonly used agents, however only two of the thirteen trials compared similar regimens: single-agent carboplatin versus combination carboplatin and paclitaxel 15,20. In the larger of the latter two trials 20, 196 patients (24%) received chemotherapy other than carboplatin alone or the combination of carboplatin and paclitaxel.

Treatment schedules varied across the trials, with the scheduled cycles of chemotherapy ranging from a low of 4 to a high of 12 14–18,20–26. One trial did not report information about cycles 19, and in one trial 24, treatment was intended to continue until progression, undue toxicity, or patient refusal.

In eight trials 14–16,20,22,23,25,26, patients were randomized to either single-agent or multi-agent chemotherapy, and in five randomized trials, single-agent chemotherapy was compared with other single-agent chemotherapy 17–19,21,24.

In five trials, only patients who responded for more than 6 months 14,15,20,22,23 after first-line treatment with a platinum-containing regimen (considered “platinum-sensitive”) were eligible to participate in the randomized studies. In four of those trials, platinum sensitivity was demonstrated beyond a 12-month period in approximately 60% 14,15,20 or 100% 22 of patients. In the remaining study, data on the persistence of platinum-sensitivity were not reported 23. In four of the five trials, second- or third-line treatment contained further platinum-containing chemotherapy; the one exception was a small study in which paclitaxel was used in one of the treatment arms 22.

In eight studies 16–19,21,23,24,25, 35%–100% of patients experienced progression less than 6 months after treatment with a platinum-containing regimen. Those patients were considered platinum-refractory or platinum-resistant. In seven of these eight studies of partial 16–19,21,24,25 or complete platinum-resistance 26, non-platinum-containing regimens were investigated. Again, the one exception was one small study in which oxaliplatin was used in one of the treatment arms 24.

In five of the trials that included patients with both platinum-sensitive and platinum-resistant disease 16–18,21,24, results were also reported separately for the two subgroups of patients.

Protocols for treatment modification included cycle delay 15–17,22–24, dose reduction 15–18,23,24,26, and use of erythropoietin 14 or granulocyte colony–stimulating factor 14,17,18,23,26. Where reported, patients were removed from the study if treatment delay was greater than 2 18 or 6 weeks 15, if adverse events occurred 15,17, 18,24, or if dose reductions fell below the minimum allowable dose 24. Patient crossover to the alternative agent, considered third-line chemotherapy, was allowed in two trials 18,22 and was not reported in the remaining trials 14–17,19–21,23–26.

The primary study endpoints were reported to be various combinations of response rate 15,18,23–26, duration of response 18, progression-free survival 14,17, 18,21, toxicity 23, and overall survival 16,17,19–22.

Seven trials reported qol as a study endpoint 14,15,17–20,24. Assessment of qol used the European Organization for Research on Cancer treatment qol questionnaire 14,15,17,18,20,24, including the ovarian cancer module 14, and a “specific checklist” that was not fully described 24. One trial reported as an abstract did not provide details on qol assessment or on qol results between treatment groups 19. The remaining trials did not report whether data on qol were collected 16,21–23,25,26.

Overall, in assessing the characteristics of the thirteen randomized trials, it was clear that the eligible trials varied widely, making it difficult to compare results across trials. Important differences in platinum sensitivity, type of chemotherapy, number of agents employed, cycles of chemotherapy, and study endpoints all contributed to inter-trial heterogeneity.

### 4.3 Trial Quality

The identified trials were non-blinded multi-centre 14,16–26 or single-centre 15 phase iii 14,16–21,23,25,26 or phase ii rcts 15,22,24. In seven trials, the randomization procedure was reported 15,16,18,20,23,24,26; the remaining trials did not report that information 14,17,19,21,22,25. Eight trials reported patient accrual with sufficient power to detect significant differences between treatment groups 14–17,20,22,23,26 at an alpha level of 0.05 15,16,20,22,23,26. Five trials did not report information on power calculations 18,19,21,24,25. One phase ii trial employed a “pick the winner design” that was sufficiently powered; however, given the small number of patients involved, a formal statistical comparison between treatment arms was not planned. That trial eventually reported statistical comparisons of primary and secondary endpoints as part of exploratory analyses 15.

Baseline characteristics were reported or observed to be generally similar across treatment groups in twelve trials 14–24,26; they were not reported in one trial 25. Statistical comparisons between baseline characteristics of the patients were reported in two trials, with no statistically significant differences being detected between treatment groups 15,21.

Across eleven trials, completeness of follow-up was reported or inferred to be greater than 80% 14–18, 20–24,26, and in two trials data on follow-up were not available 19,25. Eight trials reported data using the intent-to-treat principle 15–18,20,22,23,26. In three trials 14,21,24 where intent-to-treat information was not explicitly reported, it could be inferred from the published trial data.

In general, the methodologic quality of the trials—that is, their internal validity—was deemed to be sufficient to permit meaningful conclusions to be drawn from the results of the individual trials (external validity).

### 4.4 Outcomes

Table II shows response and survival results for the thirteen randomized trials identified in the review of the evidence.

#### 4.4.1 Survival

Survival data were reported 14–22,24,25 or extracted from survival curves 23,26 for all of the randomized trials. Survival was reported to be a primary study endpoint in six of the thirteen trials 16,17,19–22.

As Table II shows, four trials detected significant differences in survival between treatment groups 15,17, 20,22. In three trials of these trials (with 100% of the patients being platinum-sensitive), significant survival advantages were detected with combination platinum-containing chemotherapy as compared with single-agent chemotherapy 15,20,22. In the remaining trial (with a mixed platinum-sensitive and platinum-resistant patient population) 17, a statistically significant median survival advantage of 2 months was detected with liposomal doxorubicin as compared with topotecan. In that trial, no significant differences were observed in response rate or progression-free survival, and the survival curves for the treatment arms were virtually indistinguishable for approximately the first year. Shortly beyond year 1, the survival curve for liposomal doxorubicin began to show a survival benefit 17. That result could indicate that an unexplained treatment effect occurred after disease progression. One possibility is that, because liposomal doxorubicin was available only on study at the time, treatment crossover upon progression was not possible for patients in the topotecan treatment arm, but that patients in the liposomal doxorubicin arm probably would have received subsequent topotecan. The sequence of the chemotherapy delivery may perhaps account for the treatment effect; however, with limited clinical benefit and no follow-up data reported, the Gynecology Cancer dsg concluded that further confirmation of the observed results would be needed to support the superiority of one agent over another in that trial. No other survival differences were reported in the remaining nine trials 14,16,18,19,21,23–26.

Five trials reported separate subgroup analyses of survival results by platinum sensitivity 16–19,21. In the platinum-sensitive subgroups, three trials 16,18,21 reported no significant survival difference between treatment groups; one trial 17 detected a survival advantage with liposomal doxorubicin; and one trial 19 detected a survival advantage with topotecan. Gordon *et al.* 17 reported a significant survival advantage for pegylated doxorubicin over topotecan (27 months vs. 18 months, *p* = 0.017). That 9-month survival benefit seems promising, but it is based on a subgroup analysis and, again, the Gynecology Cancer dsg concluded that further confirmation of those results would be needed to establish the superiority of liposomal doxorubicin over topotecan. In the trial reported by Meier *et al.* 19, topotecan was more effective than treosulphan for patients with platinum-sensitive disease (16 months vs. 14 months, *p* = 0.0068).

For patients with platinum-refractory or platinum-resistant disease, none of the trials detected any statistically significant survival advantage with one chemotherapy agent over another.

#### 4.4.2 Progression-Free Survival

Progression-free survival was reported in eleven of the thirteen randomized trials 14–22,24,25 and was extracted from survival curves in one trial 23. The remaining trial reported median duration of response as a primary outcome of interest 26. Progression-free survival was reported to be the primary study endpoint in four of the thirteen randomized trials 14,17,18,21.

In four trials of 100% platinum-sensitive patients, a significant survival advantage was detected with combination platinum-containing chemotherapy as compared with single-agent chemotherapy with carboplatin 14,15,20 or paclitaxel 22. In one trial of non-platinum-containing chemotherapy 19, topotecan was superior to treosulphan by a span of approximately 2 months (5.4 months vs. 3.0 months, *p* < 0.001). No other statistically significant differences in progression-free survival or in median duration of response 26 were reported in the remaining trials 16–18,21,24–26.

Five trials reported subgroup analyses for progression-free survival by patient sensitivity status 16–19,21. Gordon *et al.* 17 reported a significant advantage in progression-free survival with pegylated doxorubicin over topotecan for patients with platinum-sensitive disease (6.7 months vs. 5.4 months, *p* = 0.037), but not for patients with platinum-resistant disease. In the trial reported by Meier *et al.* 19, topotecan was more effective than treosulphan for patients both with platinum-sensitive disease (6.6 months vs. 5.2 months, *p* = 0.0179) and with platinum-resistant disease (4.2 months vs. 2.2 months, *p* = 0.0279). In the two foregoing trials, the clinical benefit of the superior treatment did not exceed 2 months. In the remaining three trials 16,18,21, no significant differences were detected between treatment subgroups.

#### 4.4.3 Tumour Response

Tumour response was reported to be a primary study endpoint in six of the randomized trials 15,18,23–26. Two of these randomized trials detected statistically significant differences in overall response rate favouring carboplatin-based combination chemotherapy over single-agent carboplatin 14,15. The remaining trials reported no significant differences in response rate between treatment groups 16–26.

### 4.5 Adverse Events Associated with Chemotherapy

As Table III shows, data on adverse events varied with the treatment regimen, and although reporting differed between the studies, adverse events correlated with known toxicity profiles.

Ten trials reported statistically significant differences in adverse events by treatment group 14–18, 21–23,25,26. On average, severe adverse events, generally hematologic, were significantly associated with combination chemotherapy in trials comparing a combination regimen with single-agent chemotherapy.

The agents most commonly used were paclitaxel, carboplatin, topotecan, and pegylated doxorubicin. Adverse events associated with paclitaxel included alopecia (any grade) in 62%–100% of patients, neurotoxic effects (any grade) in 5%–42% of patients, severe leucopenia in 4%–24% of patients, and severe nausea and vomiting in 2%–6% of patients 16,18,21–22,24–26. Carboplatin was associated with low rates of severe hematologic events (typically 15% of patients or fewer), severe nausea and vomiting in approximately 10% of patients or fewer, and any grade of alopecia in 2%–25% of patients 14,15,20,23. When compared with paclitaxel or pegylated doxorubicin, topotecan was significantly associated with increased severe hematologic toxicities, and some grade of alopecia occurred in 49%–76% of patients 17,18. In the two trials that studied pegylated doxorubicin, the adverse events associated with that agent included any grade of palmar–plantar erythrodysesthesia (PPE) in approximately one half of patients and severe PPE in 23% (Gordon *et al.* 17) and 16% (O’Byrne *et al.* 21) of patients. One trial 21 also reported a significant difference in severe stomatitis in patients treated with pegylated doxorubicin as compared with those treated with paclitaxel (10% vs. 1%, *p* = 0.03).

### 4.6 Quality of Life

Six trials reported data on qol outcomes 14,15,17,18,20,24. Overall, no statistically significant differences in qol outcome were detected between treatment groups in any of these randomized trials.

Three trials reported results of subgroup analyses on specific qol outcomes 14,15,20. One trial 14 reported that symptomatic patients who received gemcitabine and carboplatin had improved global qol, including faster palliation of abdominal symptoms, than did patients treated with carboplatin alone. Another trial 15, which reported very high (>85%) non-compliance with questionnaire completion at 6 and 12 months, reported that the nausea and vomiting subscale scores were significantly higher (*p* = 0.033) with carboplatin than with the combination of carboplatin and paclitaxel. In the icon4 trial 20, the authors reported that, when the nausea and vomiting subscale was analyzed separately, qol on that subscale was significantly worse among women receiving single-agent chemotherapy than it was in women receiving multiple-agent chemotherapy (*p* = 0.0014).

## 5. DISCUSSION

The goals of therapy for patients with recurrent ovarian cancer are to improve qol and extend survival. Therefore, with regard to deriving conclusions based on the available evidence, it was important to consider the generalizability of the results, to assess the methodologic quality of the studies forming the evidentiary base, and to interpret the results in a clinically meaningful manner.

Even though the literature search uncovered thirteen rcts that investigated the role of chemotherapy in patients with recurrent ovarian cancer and prior platinum exposure, the data are incomplete. Although platinum sensitivity status is more of a continuum than a discrete event, it has an impact on patient outcomes—an impact supported by the current body of evidence. Only six of the thirteen trials included solely patients who were considered either 100% platinum-sensitive 14,15,20,22,23 or 100% platinum-resistant 26. A further five trials reported subgroup analyses by platinum sensitivity 16–19,21, but those studies were not designed to compare differences between subgroups, and their results must therefore be interpreted with caution.

In addition, study size, characteristics, and design must also be considered when interpreting the data. Eight trials provided power calculations, but four trials randomized fewer than 50 patients per arm; five trials included approximately 100 patients per arm; and the four larger studies randomized at least 150 patients per arm. Unsurprisingly, most of statistically significant results stemmed from the larger randomized trials. The characteristics of the trials varied widely, exhibiting important differences in chemotherapy regimens such as platinum versus non-platinum-containing regimens, single-agent versus combination chemotherapy, and varying lengths in the planned chemotherapy cycles, making it difficult to compare results across trials. In addition, the primary study endpoints also varied widely across the trials. These endpoints were roughly divided by response rate 15,18,23–26, progression-free survival 14,17, 18,21, and overall survival 16,17,19–22.

Despite the noted differences among the identified trials, the methodologic quality of the individual trials was reasonably high, with most trials reporting (but not statistically comparing) well-balanced baseline patient characteristics, completeness of follow-up greater than 80%, power and patient accrual sufficient to detect statistically significant differences between treatment groups, and the intent-to-treat principle. These trials were generally multicentric investigations that took place in the phase iii setting.

Overall, for patients with platinum-sensitive recurrent disease, evidence from four trials 14,15,20,22 is sufficient to conclude that platinum-containing combination chemotherapy improves response and survival outcomes as compared with single-agent carboplatin or paclitaxel. The more compelling evidence comes from one large trial 20 and one small supporting trial 15 that compared the combination of carboplatin and paclitaxel with single-agent carboplatin. The combination of gemcitabine and carboplatin also improved response and progression-free survival as compared with carboplatin alone 14. In the single-agent setting, carboplatin was not as efficacious as combination chemotherapy, but it still showed consistent efficacy across trials with a manageable toxicity profile. As part of non-platinum-containing chemotherapy, paclitaxel, topotecan, and liposomal doxorubicin showed activity in patients with platinum-sensitive disease 17,18,21; however, none of those agents has been compared in the randomized setting against platinum-based chemotherapy. In that setting, given the unexpected findings, subgroup analyses, and mixed results across trials, establishing the superiority of one non-platinum agent over another is difficult without further confirmation of results.

For patients with platinum-refractory or -resistant disease, the non-platinum-containing combination chemotherapy in three trials was no more efficacious than was the non-platinum single-agent chemotherapy; however, in terms of the number of trials, number of patients studied, and number of regimens investigated, the data are limited.

In five trials, non-platinum single-agent treatment options included paclitaxel, topotecan, liposomal doxorubicin, or treosulphan. One trial 17 reported a 2-month statistically significant difference in median survival with liposomal doxorubicin in a mixed platinum-sensitive patient population; however, the limited clinical benefit, coupled with unexpected findings, subgroup analyses, and a lack of confirmation of results leads to a conclusion of uncertain superiority for the agents being compared. On the basis of one trial 19, topotecan appears to be more effective than treosulphan in extending progression-free survival, but that result does little to inform the clinical scenario in the context of current clinical practice. It should be noted that the survival benefit of any one chemotherapy agent over another did not exceed 3 months in any of the randomized trials.

Thus, for patients with platinum-refractory or -resistant disease, the data provide little definitive comment on the efficacy of combination chemotherapy as compared with single-agent chemotherapy or on the efficacy of any single agent over another.

## 6. CONCLUSIONS

Given the available data, it would seem reasonable to conclude that, as long as patients continue to respond to platinum-based chemotherapy with no contraindications, then combination platinum-based chemotherapy should be considered for this patient population. This conclusion is based on the improved survival benefits, but recognizes the increase in adverse events.

If combination therapy is not indicated (because of toxicity or otherwise), then a single platinum agent should be considered if indicated. Single-agent carboplatin has been effective in this patient population, and it has a manageable toxicity profile. Patients could continue to receive platinum-based chemotherapy across multiple lines, if need be, for as long as they remain sensitive to platinum-containing chemotherapy.

If platinum-containing chemotherapy is not being considered, then monotherapy with a non-platinum agent should be considered on the basis of patient preference, toxicity profile, ease of administration, and availability. Single-agent paclitaxel, topotecan, or pegylated liposomal doxorubicin have demonstrated activity in this patient population and are reasonable treatment options.

For patients who do not respond to platinum-based chemotherapy, then treatment decisions should be based on patient preference, toxicity profile, ease of administration, and availability. The use of combination chemotherapy does not appear to be advantageous in this group of patients, and no evidence either supports or refutes the use of multiple lines of chemotherapy in patients with platinum-resistant recurrences. Single-agent paclitaxel, topotecan, or pegylated liposomal doxorubicin have demonstrated activity in this patient population and are reasonable treatment options.

Platinum-sensitivity status in these patients should be regarded as a continuum rather than a discrete outcome. It is therefore recognized that each patient needs to be individually assessed to determine optimal therapy in terms of recurrence, sensitivity to platinum, toxicity, ease of administration, and patient preference. Participation in randomized trials should be encouraged for all patients, where appropriate.

Further research from randomized trials is needed to determine the optimum chemotherapeutic regimen for patients with both platinum-sensitive and platinum-resistant recurrent ovarian cancer. The roles of further cytoreductive surgery and carboplatin desensitizing agents remain to be determined. Currently, studies planned or underway include the Southwest Oncology Group S0200 phase iii trial of carboplatin with or without pegylated doxorubicin in platinum-sensitive disease, the calypso study (a Gynaecologic Cancer Intergroup study) of carboplatin and paclitaxel versus carboplatin and liposomal doxorubicin in platinum-sensitive recurrence, and another Intergroup study (icon6, led by the U.K. Medical Research Council) in patients with platinum-sensitive disease, which will explore the addition of a targeted molecular agent (AZD2171, against vascular endothelial growth factor) to carboplatin and paclitaxel therapy. The results of ongoing trials should help to determine the most appropriate treatment options for patients with recurrent epithelial ovarian cancer.

## 7. INTERNAL REVIEW OF THE PRACTICE GUIDELINE REPORT

### 7.1 RAP Feedback

Before external review, the draft report was reviewed by the pebc rap, which consists of 2 members, including an oncologist with expertise in clinical and methodology issues.

One major issue raised by the rap was that the link between the evidence and the recommendations for platinum-resistant or -refractory patients was confusing to follow. The rap commented that although two trials appeared to show differences in survival, the relevant information did not appear to factor into the formulation of the practice recommendation. Related to this issue was the comment that greater discussion was required concerning the dsg’s interpretation of results from one trial in which methodologic issues were raised about the crossing of survival curves, but in which was also detected a significant survival difference between treatment groups. The rap commented that the dsg had not adequately justified its statement regarding the methodologic concerns potentially affecting the survival outcomes in that trial.

As part of minor comments, suggestions about the structure and format of the report were also offered. The rap found that the text-dense format of the Trial Characteristics and Trial Quality sections made it difficult for readers to link the various elements and to interpret the findings in a meaningful manner. One rap member felt that use of subheadings could be helpful, and one suggested that the information might be better presented in tables.

As a final comment, the rap noted that a reference in the Introduction to the case fatality ratio was unclear about whether that value came from a separate reference or was calculated from the incidence and mortality data presented in the same sentence. The rap suggested that the dsg reword or clarify.

### 7.2 Modifications

In response to the rap’s comments, the Gynecology Cancer dsg addressed the presentation of the link between the evidence and the recommendations in the Survival, Discussion, and Key Evidence sections. The primary issue was that results from the trial by Gordon *et al.,* which detected an overall 2-month survival benefit with pegylated doxorubicin over topotecan was not reflected in the recommendations where liposomal doxorubicin, topotecan, and paclitaxel were all listed as reasonable treatment options. The findings from that trial were unexpected, because no significant differences in response rate or progression-free survival had been detected between the treatment arms, and the survival curves for the treatment arms were virtually indistinguishable for approximately the first year. The trial then went on to show a 2-month survival benefit shortly beyond year 1 that was even more pronounced in the subgroup analysis of platinum-sensitive patients (9-month improvement in median survival).

The Survival section hypothesized that a treatment effect may have occurred after disease progression, one possibility being that, because liposomal doxorubicin was available only on study at the time, treatment crossover upon progression was not possible for patients in the topotecan treatment arm, but patients in the liposomal doxorubicin arm were likely to have received subsequent topotecan. Perhaps the sequence of the chemotherapy delivery can account for the treatment effect. However, with the unexpected findings, the limited clinical benefit outside of subgroup analysis, and no follow-up data reported, that trial would need further confirmation of results before the Gynecology Cancer dsg could conclude superiority of one agent over another.

In the second trial in which the superiority of topotecan over treosulphan in progression-free survival was detected, the results from that trial did little to inform the clinical picture, given that treosulphan was not being recommended as one of the three single agents being considered as reasonable treatment options. Potentially confusing subgroup results from that trial were removed from Table II, and the description of the results was revised to improved clarity.

To improve clarity in the Results section, summary sentences were added to the Trial Characteristics and Trial Quality subsections: one sentence provides an overall assessment of the relative homogeneity or heterogeneity of the identified trials, and the other comments on external validity through an assessment of the internal validity of the trials. The text in these sections was also divided into smaller paragraphs to improve readability.

Finally, where the Introduction referred to the potentially confusing case fatality ratio, the wording was changed to a ratio of mortality to incidence.

## 8. EXTERNAL REVIEW OF THE PRACTICE GUIDELINE REPORT

### 8.1 Practitioner Feedback Survey

The systematic review and practice guideline were distributed for review and feedback to practitioners throughout Ontario, Canada, in accordance with the practice guidelines development cycle 12. A sample of 170 practitioners in Ontario received the survey, which consisted of items evaluating the methods, results, and interpretive summary used to inform the draft recommendations and of questions about whether the draft recommendations should be approved as a practice guideline. Written comments were invited. The practitioner feedback survey was mailed on September 18, 2006, and a complete repeat mailing was sent thereafter. The Gynecology Cancer dsg reviewed the results of the survey.

### 8.2 Survey Results

From among the 170 surveys sent, 85 responses were received. Responses include returned completed surveys, plus telephone, fax, and e-mail responses. Of the practitioners who responded, 22 indicated that the report was relevant to their clinical practice, and they completed the survey. Table IV summarizes key results.

Of the respondents, 7 provided written comments. In general, the comments were highly positive and accorded with the conclusions derived by the Gynecology Cancer dsg. Five practitioners commented favourably on the quality and development of the series overall, although one practitioner asked why the dsg would undertake a guideline given the level of evidence available for analysis. One practitioner commented that highly motivated patients with platinum-resistant disease often receive more than one line of chemotherapy. One practitioner suggested that a comment be added about treatment in platinum-sensitive patients after third-line chemotherapy. Two practitioners commented that addressing related topics such as carboplatin desensitization or further cytoreductive surgery would be helpful.

### 8.3 Action Taken

On the basis of the survey results, which were supportive of the guideline, the Gynecology Cancer dsg concluded that no substantive revisions to the document were needed.

The comment regarding the body of evidence in this treatment setting makes a valid point. However, the Gynecology Cancer dsg agreed that that it was possible and necessary to derive meaningful conclusions from the available randomized evidence. The need for further research on this topic area is well recognized. The authors also recognize that the ideal number of regimens is not well informed by the evidence, but strategies for the management of recurrent ovarian cancer is the focus of another guideline in development by the Gynecology Cancer dsg. The impact of related issues such as further cytoreductive surgery and the use of carboplatin desensitizing agents are of interest, but are not a focus of the present report. A sentence regarding future research in these areas was added.

The present report reflects the integration of the feedback obtained through the external review process with the final approval given by the rap and the Gynecology Cancer dsg. Updates of the report will be issued as new evidence informing the question of interest emerges.

## 9. CONFLICT OF INTEREST

Members of the Gynecology Cancer dsg disclosed potential conflict of interest information. No conflicts were reported.

## Figures and Tables

**TABLE I:** Literature search results and selected trial characteristics

			Treatment regimen			Platinum-sensitive patients (%)		
Reference	Patients (*n*)	Agent	Dose	Day	Cycles (planned)	<6 mo.	≥6 mo.	ct line
Pfisterer *et al.*, 2005^14^	178	Carboplatin	auc=5	1	6	0	100^a^	2
ncic ov15	178	Carboplatin/gemcitabine	auc=4 1000 mg/m^2^	1 1+8	6 6	0	100 a	2
Gonzalez–Martin *et al.*, 2005 15	40	Carboplatin	auc=5	1	6–9	0	100 a	2–3
geico	41	Carboplatin/paclitaxel	auc=5 175 mg/m^2^	1 1	6–9 6–9	0	100^a^	2–3
Buda *et al.*, 2004^16^	106	Paclitaxel	175 mg/m^2^	1	4–6	75	25	2
gono/ior	106	Paclitaxel/epirubicin	175 mg/m^2^ 80 mg/m^2^	1 1	4–6 4–6	73	27	2
Gordon *et al.*, 2004^17^	239	Pegylated doxorubicin	50 mg/m^2^	1	12	54	46	2
Doxil 30-49	235	topotecan	1.5 mg/m^2^	1–5	12	53	47	2
ten Bokkel Huinink *et al.*, 2004^18^	112	Paclitaxel	175 mg/m^2^	1	12	52	48	2
itsg	114	Topotecan	1.5 mg/m^2^	1–5	12	54	46	2
Meier *et al.*, 2004 19,b	179	Treosulfan	7.0 g/m^2^	nr	nr	36	64	2–3
ago	178	Topotecan	1.5 mg/m^2^	1–5	nr	34	66	2–3
Parmar *et al.*, 2003 20	410	Carboplatin^c^	auc≥5	1	6–8	0	100^a^	2^d^
icon4/ago	392	Carboplatin/paclitaxel^c^	auc≥5 ≥175 mg/m^2^	1 1	6–8 6–8	0	100^a^	2^d^
O’Byrne *et al.*, 2002 21,b	107	Pegylated doxorubicin	50 mg/m^2^	1	nr	60	40	2
	107	Paclitaxel	175 mg/m^2^	1	nr	63	37	2
Cantu *et al.*, 2002^22^	50	Paclitaxel	175 mg/m^2^	1	≥6	0	100^e^	2
	47	Cyclophosphamide/doxorubicin/cisplatin	500 mg/m^2^ 50 mg/m^2^ 50 mg/m^2^	1 1 1	≥6 ≥6 ≥6	0	100^e^	2
Bolis *et al.*, 2001^23^	95	Carboplatin	300 mg/m^2^	1	5	0	100	2
	95	Carboplatin/epirubicin	300 mg/m^2^ 120 mg/m^2^	1 1	5 5	0	100	2
Piccart *et al.*, 2000^24^	41	Paclitaxel	175 mg/m^2^	1	6^f^	76	24	2–3
	45	Oxaliplatin	130 mg/m^2^	1	4^f^	71	29	2–3
Torri *et al.*, 2000^25^,^b^	116	Paclitaxel	175 mg/m^2^	1	4–6	≥50^g^	≤50^g^	2
	118	Paclitaxel/doxorubicin	175 mg/m^2^ 80 mg/m^2^	1 1	4–6 4–6	≥50^g^	≤50^g^	2
Bolis *et al.*, 1999^26^	41	Paclitaxel	175 mg/m^2^	1	5	100	0	2
	40	Paclitaxel/epirubicin	150 mg/m^2^ 120 mg/m^2^	1 1	5 5	100	0	2

a Approximately 60% of patients were platinum-sensitive beyond 12 months.

b Abstract data from conference proceedings.

c The authors reported that 24% of patients received chemotherapy other than carboplatin or paclitaxel and carboplatin.

d Among these patients, 8% received third- or greater-line chemotherapy.

e In this trial, 100% of patients were platinum-sensitive beyond 12 months.

f Actual median number of cycles delivered.

g Median time from the end of first-line chemotherapy to trial randomization was 5 months, with a range of 3–12 months.

ct = chemotherapy; ncic ov15 = National Cancer Institute of Canada ov15 trial; auc = area under curve; geico = Grupo Espanol de Investigacion en Cancer de Ovario; gono/ior = Gruppo Oncologico Nord Ovest/Istituto Oncologico Romagnolo; Doxil 30-49 = Doxil Study 30-49; itsg = International Topotecan Study Group; ago = Arbeitsgemeinschaft Gynaekologische Onkologie.

**TABLE II:** Response rates and survival outcomes

				Response and survival outcomes^a^			
				Response (%)		Survival (mo.)	
Reference	Patients (*n*)	Treatment group	Platinum- sensitive (%)(≥6 mo.)	cr	or (pr+cr)	pfs	Median
Pfisterer *et al.*, 2005^14^	178	Carboplatin	100	6	31	6	17
ncic ov15	178	Carboplatin/ gemcitabine	100	15 ***p*<0.01**	47	9 ***p*<0.01** **hr=0.72** **(0.58–0.90)**	18
Gonzalez–Martin *et al.*, 2005^15^	40	Carboplatin	100	20	50	8	17
geico	41	Carboplatin/ paclitaxel	100	27	76 ***p*=0.02**	11 ***p*=0.02** **hr=0.54** **(0.32–0.92)**	Not reached ***p*=0.002** **hr=0.31** **(0.14–0.68)**
Buda *et al.*, 2004^16^	106	Paclitaxel	25	20^b^	47^b^	6	14
gono/ior	106	Paclitaxel/ epirubicin	27	15^b^	37^b^	6	12
Gordon *et al.*, 2004^17^ Doxil 30-49	239	Pegylated doxorubicin	46	4	20	4	15
	235	Topotecan	47	5	17	4	13 ***p*=0.038** **hr=1.23** **(1.01–1.50)**
ten Bokkel Huinink *et al.*, 2004^18^	114	Paclitaxel	48	3	13	3	12
itsg	112	Topotecan	46	5	21	4	15
Meier *et al.*, 2004^19^	179	Treosulfan	64	nr	nr	3	nr
ago	178	Topotecan	66	nr	nr	5 ***p*<0.001**	nr
Parmar *et al.*, 2003^20^	410	Carboplatin^c^	100	nr	54^b^	9	24
icon4/ago	392	Carboplatin/ paclitaxel^c^	100	nr	66^b^	12 ***p*=0.0004** **hr=0.76** **(0.66–0.89)**	29 ***p*=0.02** **hr=0.82** **(0.69–0.97)**
O’Byrne *et al.*, 2002^21^	107	Pegylated doxorubicin	40	2	19	4	11
	107	Paclitaxel	37	6	23	5	13
Cantu *et al.*, 2002^22^	50	Paclitaxel	100	17	45	9	26
	47	cap	100	30	55	16 ***p*=0.038** **hr=0.60** **(0.37–0.97)**	35 ***p*=0.043** **hr=0.58** **(0.34–0.98)**
Bolis *et al.*, 2001^23^	95	Carboplatin	100	35	55	~15	~23
	95	Carboplatin/ epirubicin	100	30	58	~18	~28
Piccart *et al.*, 2000^24^	41	Paclitaxel	24	0	17	3	9
	45	Oxaliplatin	29	0	16	3	10
Torri *et al.*, 2000^25^	116	Paclitaxel	≤50	nr	54	8	14
	118	Paclitaxel/ doxorubicin	≤50	nr	52	7	12
Bolis *et al.*, 1999^26^	41	Paclitaxel	0	7	17	6^d^	~9
	40	Paclitaxel/ epirubicin	0	13	34	10^d^	~14

a Data are rounded and only statistically significant differences between treatment groups are shown in boldface.

b Based on evaluable patients.

c The authors reported that 24% of patients received chemotherapy other than carboplatin or paclitaxel and carboplatin.

d Median duration of response.

cr = complete response; or = objective response; pr = partial response; pfs = progression-free survival; ncic ov15 = National Cancer Institute of Canada ov15 trial; hr = hazard ratio; geico = Grupo Espanol de Investigacion en Cancer de Ovario; gono/ior = Gruppo Oncologico Nord Ovest/Istituto Oncologico Romagnolo; Doxil 30-49 = Doxil Study 30-49; itsg = International Topotecan Study Group; ago = Arbeitsgemeinschaft Gynaekologische Onkologie; icon = International Collaborative Ovarian Neoplasm Group; nr = not reported; cap = cyclophosphamide/doxorubicin/cisplatin; ~ = approximate values extracted from survival curves.

**TABLE III:** Patients with grade 3 or 4 adverse events during chemotherapy

			Adverse events (%)									
			Grade 3 or 4						All grades			
Reference	Patients (*n*)	Treatment group	Anemia	Leucopenia	Neutropenia	Thrombocytopenia	Nausea	Vomiting	Diarrhea	Constipation	Neurotoxicity	Alopecia
Pfisterer *et al.*, 2005^14^	178	Carboplatin	8	nr	12	12	2	2	0	nr	5	2^a^
ncic ov15	178	Carboplatin/ gemcitabine	27 ***p*<0.01**	nr	70 ***p*<0.01**	35 ***p*<0.01**	4	3	2	nr	5	14^a^ ***p*<0.01**
Gonzalez–Martin *et al.*, 2005^15^	40	Carboplatin	15	3	10	13	0	10	3^b^	8^b^	0^b^	18^b^
geico	38	Carboplatin/ paclitaxel	5	5	18	3	0	3	3^b^	8^b^	24^b^ ***p*<0.009**	87^b^ ***p*=0.001**
Buda *et al.*, 2004^16^	99	Paclitaxel	5	9	18	1	6		nr	nr	14^c^	69^d^
gono/ior	99	Paclitaxel/ epirubicin	3	19	37 ***p*=0.01**	1	11		nr	nr	12^c^	62^d^
Gordon *et al.*, 2004^17^	239	Pegylated doxorubicin	5	10	12	1	nr	nr	nr	nr	nr	16
Doxil 30-49	235	Topotecan	28 ***p*<0.001**	50 ***p*<0.001**	77 ***p*<0.001**	34 ***p*<0.001**	nr	nr	nr	nr	nr	49 ***p*=0.007**
ten Bokkel Huinink *et al.*, 2004^18^	114	Paclitaxel	6	21	52	3	2	3	1^d^	0^d^	16	93
itsg	112	Topotecan	41	85	95 ***p*<0.01**	50 ***p*<0.01**	10	10	6^d^	5^d^	1	76
Meier *et al.*, 2004^19^	179	Treosulfan	1	nr	5	2	nr	nr	nr	nr	nr	nr
ago	178	Topotecan	4	nr	47	7	nr	nr	nr	nr	nr	nr
Parmar *et al.*, 2003^20^	410	Carboplatin	46^e^				40^b^	nr	nr	1^b^	25^b^	40^b^
icon4/ago	392	Carboplatin/ paclitaxel	29^e^				35^b^	nr	nr	20^b^	86^b^	35^b^
O’Byrne *et al.*, 2002^21^	107	Pegylated doxorubicin	2	6	7	nr	7	9	nr	nr	13^f^	44
	107	Paclitaxel	4	8	13	nr	2	2	nr	nr	42^f^ ***p*=0.002**	88 ***p*=0.002**
Cantu *et al.*, 2002^22^	47	Paclitaxel	nr	4	13	0	17^c^		nr	nr	11	87
	47	cap	nr	34 ***p*=0.001**	36 ***p*=0.009**	13 ***p*=0.012**	51^c^ ***p*=0.004**		nr	nr	6 ***p*=0.002**	60 ***p*=0.01**
Bolis *et al.*, 2001^23^	95	Carboplatin	10	13	nr	20	3		nr	nr	nr	5^g^
	95	Carboplatin/ epirubicin	25 ***p*<0.05**	53 ***p*<0.05**	nr	64 ***p*<0.05**	13 ***p*<0.05**		nr	nr	nr	88^g^***p*<0.01**
Piccart *et al.*, 2000^24^	41	Paclitaxel	2	nr	22	0	2	2	0^g^	nr	7^g^	nr
	45	Oxaliplatin	2	nr	0	4	4	7	4^g^	nr	9^g^	nr
Torri *et al.*, 2000^25^	116	Paclitaxel	nr	7	nr	nr	nr	nr	nr	nr	19^c^	nr
	118	Paclitaxel/ doxorubicin	nr	24 ***p*<0.05**	nr	nr	nr	nr	nr	nr	19^c^	nr
Bolis *et al.*, 1999^26^	41	Paclitaxel	12	24	nr	2	2		nr	nr	15^c^	100
	40	Paclitaxel/ epirubicin	30 ***p*=0.04**	45 ***p*=0.05**	nr	25 ***p*=0.003**	8		nr	nr	5^c^	100

a Grade 2 toxicity.

b Grades 2–4 toxicity combined.

c Grades 2–3 toxicity combined.

d Grades 3–4 toxicity combined.

e Hematologic toxicity leading to treatment modification or interruption.

f Paresthesia.

g Grade 3 toxicity.

ncic ov15 = National Cancer Institute of Canada ov15 trial; nr = not reported; geico = Grupo Espanol de Investigacion en Cancer de Ovario; gono/ior = Gruppo Oncologico Nord Ovest/Istituto Oncologico Romagnolo; Doxil 30-49 = Doxil Study 30-49; itsg = International Topotecan Study Group; ago = Arbeitsgemeinschaft Gynaekologische Onkologie; icon = International Collaborative Ovarian Neoplasm Group; cap = cyclophosphamide/doxorubicin/cisplatin.

**TABLE IV:** Practitioner responses to eight items on the practitioner feedback survey

Item	Strongly agree or agree	Responses [*n* (%)] Neither agree nor disagree	Strongly disagree or disagree
The rationale for developing a clinical practice guideline, as stated in the “Choice of Topic” section of the report, is clear.	19 (90.5)	1 (4.8)	1 (4.8)
There is a need for a clinical practice guideline on this topic.	19 (86.4)	1 (4.5)	2 (9.1)
The literature search is relevant and complete.	19 (90.5)	1 (4.8)	1 (4.8)
The results of the trials described in the report are interpreted according to my understanding of the data.	20 (90.9)	1 (4.5)	1 (4.5)
The draft recommendations in this report are clear.	20 (90.9)	1 (4.5)	1 (4.5)
I agree with the draft recommendations as stated.	20 (90.9)	0 (0.0)	2 (9.1)
This report should be approved as a practice guideline.	17 (81.0)	2 (9.5)	2 (9.5)

	Very likely or likely	Unsure	Not at all likely or unlikely

If this report were to become a practice guideline, how likely would you be to make use of it in your own practice?	20 (95.3)	0 (0.0)	1 (4.8)
